# Characteristics of Nodular Goitre and Its Correlation With Papillary Thyroid Carcinoma: A Cross-Sectional Study

**DOI:** 10.7759/cureus.74780

**Published:** 2024-11-29

**Authors:** Meng Wang, Shaogang Ma, Guangwei Jiang, Delian Du

**Affiliations:** 1 Department of Endocrinology and Metabolism, Linping Campus, The Second Affiliated Hospital, Zhejiang University School of Medicine, Hangzhou, CHN; 2 Department of Endocrinology and Metabolism, The People’s Hospital of Laibin, Laibin, CHN; 3 Department of Intensive Care Unit, The People's Liberation Army's 903rd Hospital, Hangzhou, CHN

**Keywords:** cancer biomarker, clinical analysis, nodular goitre, papillary thyroid carcinoma, thyroid-stimulating hormone

## Abstract

Objective

To investigate the clinical characteristics of nodular goitre (NG) and the relationship between NG and papillary thyroid carcinoma (PTC).

Methods

A total of 282 consecutive patients suspicious for thyroid cancer were enrolled. All the patients underwent surgical resection of the thyroid. Laboratory tests, ultrasonography, and pathology data were compared between patients with and without NG.

Results

Of the 282 patients who underwent surgery, 124 were diagnosed with PTC by pathology. NG accounted for 48.9% (138/282) of all patients, with a male-to-female ratio of 1:3. The rates of PTC and haemorrhage in patients with NG were 23.2% (31/138) and 29.0% (40/138), respectively, both of which were greater than those in patients without NG (p < 0.001). The levels of thyroid-stimulating hormone (TSH) and carcinoembryonic antigen (CEA) in patients with PTC were 2.2 µIU/mL (min, 0.4; max, 109.8) (p = 0.04) and 1.16 ng/mL (0, 2.17, p25, p75) (p < 0.001), respectively, both of which were greater than those in patients with NG. However, the levels of thyroglobulin, thyroglobulin antibodies, and thyroid peroxidase antibodies were not significantly different.

Conclusions

The proportion of NG in thyroid surgery is very high, and NG carries a high risk of PTC, suggesting that elevated TSH and CEA are closely related to PTC.

## Introduction

Thyroid carcinoma is the most common endocrine and neck tumour. The incidence of thyroid carcinoma has increased in recent years. Although thyroid carcinoma accounts for only 1% of all cancers, papillary thyroid carcinoma (PTC) represents approximately 90% of all thyroid cancers [1]. Thyroid cancer was responsible for 586,000 cases worldwide, ranking ninth in incidence in 2020 [2]. The overdiagnosis of thyroid conditions through ultrasonography and fine-needle aspiration biopsy may contribute to the increase in PTC.

Overall, there are many risk factors for nodular goitre (NG), and its pathogenesis is unknown. The pathogenesis is not only inherited but also caused by somatic deleterious mutations [3]. The thyroid gland has been enlarged for a long time and is accompanied by fibrous tissue. On the other hand, excessive hyperplasia of thyroid follicles leads to the transformation of normal thyroid tissue into NG. The causative factors for NG are similar to those for thyroid carcinoma. The rate of thyroid carcinoma in NG is approximately 5% [4]. NG includes both multinodular goitre (MNG) and solitary nodular goitre (SNG). Some studies have shown that the risk of thyroid carcinoma in MNG and SNG is similar [5].

The existing evidence indicates that NG is different from other thyroid nodules and can progress to PTC. In recent years, studies have shown that PTC is related to radiation exposure, iodine intake, genetic mutations, and several metabolic factors. However, no effective or accurate biomarkers are available for the early diagnosis of NG and thyroid carcinoma. The purpose of this study was to explore the correlation between NG and PTC, as well as the value of routine clinical markers in reflecting PTC.

## Materials and methods

The present retrospective study was conducted at Linping Campus, The Second Affiliated Hospital, Zhejiang University School of Medicine, Hangzhou, China, from January 2022 to December 2022. It was approved by the Ethics Committee of the hospital (No. 2021041).

The electronic medical records were used to select suitable patients who were older than 18 years, had undergone thyroid ultrasonography and pathology, and were diagnosed with thyroid cancer based on thyroid ultrasonography. A total of 393 initial subjects (101 males and 292 females) were screened. Individuals with missing data, such as general clinical data, thyroid function panels (seven items), tumour markers, or blood lipids, were excluded from the study. In the end, 282 subjects (65 males and 217 females) qualified, including 124 subjects with PTC and 158 subjects without PTC.

All thyroid ultrasound examinations for the study subjects were conducted and reported by professional ultrasonographers from the Ultrasound Department of our hospital. The thyroid pathological tissue examinations, including tissue slicing, staining, and issuance of pathological reports, were all carried out by professional pathologists in our hospital's pathology laboratory. Additionally, all study subjects underwent seven thyroid function tests, thyroglobulin (Tg) tests, tumour marker tests, and lipid tests, all of which were completed and reported by professional laboratory technicians in our hospital's testing laboratory. The demographic variables and levels of thyroid function and tumour markers were recorded. The patients were examined by thyroid ultrasound first, followed by thyroid surgery, and the thyroid carcinoma, NG, or other benign thyroid tumours were pathologically confirmed.

This study aims to investigate the probability of PTC occurring in patients with NG, and further explore the clinical characteristics of this population.

Statistical analysis

Statistical analyses were conducted with SPSS for Windows, Version 16.0 (Released 2007; SPSS Inc., Chicago, IL, USA). Continuous data are shown as the mean ± SD or median (min-max), and categorical variables, such as sex, the incidence of only-NG, NG coexistent thyroid benign tumour (NG-nonPTC), NG coexistent PTC (NG-PTC), and the rate of calcification or haemorrhage, are presented using percentages. Categorical data were compared using the Chi-square test. Continuous data were calculated using an independent-sample t-test and analysis of variance (ANOVA). Continuous variables were log-transformed if they were not normally distributed, and the Kruskal-Wallis H test was used if the data were not normally distributed.

## Results

A total of 282 patients (65 males and 217 females) diagnosed with thyroid cancer by thyroid ultrasonography were enrolled in the study. All patients underwent total or subtotal thyroidectomy. A total of 124 patients were diagnosed with PTC, and 158 were diagnosed without PTC (107 with NG, 20 with Hashimoto thyroiditis, 18 with thyroid adenoma, and three with follicular thyroid hyperplasia). In other words, the 282 subjects included 138 with NG and 144 without NG (non-NG). Ultrasonography and biomarker levels were compared between the 138 NG patients and the 144 non-NG patients. The 138 NG patients were divided into three groups: only NG patients (NG group, n = 51), NG patients with PTC (NG-PTC group, n = 31), and NG patients with benign thyroid tumours (NG-nonPTC group, n = 56).

The ratio of males to females was 1:3.45 for the 138 patients with NG. Similarly, there were more females than males with PTC, and the ratio of females to males was 3:1. As shown in Table 1, the participants were divided into two different groups: 138 (48.9%) with NG and 144 (51.1%) without NG. The median age for all the patients was 48.2 years (19-79 years); the median age for patients with NG was 50.2 years (25-79 years), and the median age for patients without NG was 46.2 years (19-79 years). The age of patients in the NG group was significantly greater than that of patients in the non-NG group (p = 0.009). The percentage of NG patients increased with age; however, this increase was not statistically significant (p = 0.12). Thirty-one patients with calcification were observed. Calcification occurred in 24 (17.4%) NG patients. There was a significant difference in calcification between patients with NG and patients without NG (p = 0.001). In contrast, patients were more likely to experience haemorrhage. Moreover, haemorrhage was significantly more common in the NG group than in the non-NG group (p < 0.001). There were 40 haemorrhages in patients with NG. Overall, the rate of haemorrhage (29.0%) was greater than the rate of calcification (17.4%) in the NG group. The level of thyrotropin, also known as thyroid-stimulating hormone (TSH), in NG patients was 1.96 (0.1-11.0) µIU/mL, lower than that in non-NG patients, who had a TSH level of 2.0 (0-109.8) µIU/mL (p = 0.009). The serum level of carcinoembryonic antigen (CEA) in NG patients was 0 (0-0.66) ng/mL. However, the level of Tg in NG patients was 51.9 ± 11.6 ng/mL, which was higher than that in non-NG patients, whose Tg level was 38.4 ± 7.6 ng/mL (p = 0.022). The median age for patients without NG was 46.9 years (23-68 years). The percentage of NG patients increased with age; however, an upward trend was not found in patients with non-NG. The peak age of non-NG patients was 40-49 years. There was no significant relationship between age and the incidence of non-NG (p = 0.40). Calcification occurred in seven (4.9%) non-NG patients, which was lower than that of NG patients (17.4%), and there were six haemorrhages in patients without NG. The level of TSH in non-NG patients was 2.0 (0-109.8) µIU/mL, which was higher than that in NG patients, whose TSH level was 1.96 (0.1-11.0) µIU/mL (p = 0.009). The serum level of CEA in non-NG patients was 1.25 (0-2.99) ng/mL. However, the level of Tg in non-NG patients was 38.4 ± 7.6 ng/mL, which was lower than that in NG patients, whose Tg level was 51.9 ± 11.6 ng/mL (p = 0.022). Further subgroup analysis by sex revealed that NG patients had lower levels of TSH, CEA, and Tg. However, there was no significant difference between NG and non-NG patients. The higher levels of TSH and serum CEA in the non-NG group may indicate that the non-NG group had a greater rate of thyroid carcinoma.

**Table 1:** General clinical characteristics of 282 patients *p < 0.05, **p < 0.001 TSH: median (min-max), CEA: median (p25-p75), were compared using the Kruskal-Wallis H test NG, nodular goitre: non-NG, without nodular goitre; TG, triglycerides; TC, total cholesterol; LDL, low-density lipoprotein; TSH, thyroid-stimulating hormone; Tg, thyroglobulin; CEA, carcinoembryonic antigen, PTC, papillary thyroid carcinoma; TPOAb: thyroid peroxidase antibodies; TGAb: thyroglobulin antibodies

Variables	Total (n = 282)	NG (n = 138)	Non-NG (n = 144)	p-value
Male/female	65/217	31/107	34/110	0.817
Age (years)*	48.2 ± 12.8	50.2 ± 12.6	46.2 ± 12.7	0.009
Height (cm)	161.8 ± 7.4	161.4 ± 7.3	162.1 ± 7.5	0.956
Weight (kg)	65.6 ± 10.7	65.3 ± 10.0	65.8 ± 11.3	0.262
TG (<1.7 mmol/L)	1.4 ± 0.8	1.4 ± 0.8	1.4 ± 0.8	0.395
TC (3.0-5.7 mmol/L)	4.6 ± 0.9	4.7 ± 0.9	4.5 ± 0.8	0.232
LDL (1.89-4.21 mmol/L)	3.0 ± 0.6	3.1 ± 0.7	3.0 ± 0.6	0.174
TSH (0.35-4.94 µIU/mL)*	2.0 (0-109.8)	1.96 (0.1-11.0)	2.0 (0-109.8)	0.009
FT3 (2.43-6.01 pmol/L)	5.5 ± 1.0	5.6 ± 1.3	5.4 ± 0.7	0.356
FT4 (9.01-19.05 pmol/L)	18.0 ± 5.1	17.9 ± 5.9	18.2 ± 4.1	0.542
Tg (1.28-50 ng/mL)				
Total*	46.3 ± 10.2	51.9 ± 11.6	38.4 ± 7.6	0.022
Male	49.4 ± 12.2	73.5 ± 16.2	21.7 ± 2.7	0.542
Female	45.4 ± 9.5	46.2 ± 10.1	44.2 ± 8.6	0.453
TGAb (<4.11 IU/mL)	74.7 ± 7.3	25.0 ± 5.9	29.5 ± 8.9	0.484
TPOAb (<5.61 IU/mL)	30.3 ± 6.8	23.0 ± 4.2	38.2 ± 8.8	0.268
PTC**	124 (44.0%)	31 (22.5%)	92 (63.9%)	<0.001
Calcification*	31 (11.0%)	24 (17.4%)	7 (4.9%)	0.001
Haemorrhage**	46 (16.3%)	40 (29.0%)	6 (4.2%)	<0.001
CEA (ng/mL)				
Total**	0 (0-1.37)	0 (0-0.66)	1.25 (0-2.99)	<0.001
Male	0 (0-2.21)	0 (0-0)	1.9 (0.79-3.35)	0.272
Female	0 (0-1.1)	0 (0-0.79)	1.07 (0-1.83)	0.257

As shown in Table 2, the rate of low-level CEA (74.7%) in the NG group was greater than that in the high-level CEA group (18.9%), and the rate of non-NG in the low-level CEA group (32.0%) was lower than that in the high-level CEA group (46.4%) (p < 0.001). However, there was no continuous increase in the percentage of NG-free samples, nor a continuous decrease with increasing CEA levels. Similarly, the differences in TSH and Tg between the NG and non-NG groups were significant (p < 0.05). This indicates that the rates of non-NG in the high-level TSH and Tg groups were greater than those in the NG group.

**Table 2:** Difference in the rate of nodular goitre and non-nodular goitre at different levels of TSH, CEA, and Tg. *p < 0.05, **p < 0.001 TSH: median (min-max), CEA: median (p25-p75), were compared using the Kruskal-Wallis H test TSH, thyroid-stimulating hormone; Tg, thyroglobulin; CEA, carcinoembryonic antigen

Variables	Reference range	NG (n = 138)	Non-NG (n = 144)	p-value
TSH (µIU/mL)*	0-1.5	56 (58.9%)	39 (41.1%)	0.031
	1.5-2.3	37 (39.8%)	56 (60.2%)	
	2.3-109.8	45 (47.9%)	49 (52.1%)	
CEA (ng/mL)**	0-0.65	103 (69.1%)	46 (30.9%)	<0.001
	0.66-1.33	9 (22.5%)	31 (77.5%)	
	1.37-9.48	26 (28.0%)	67 (72.0%)	
Tg (ng/mL)*	0-4.48	59 (67.8%)	28 (32.2%)	0.003
	4.66-22.05	38 (38.0%)	62 (62.0%)	
	22.8-500.0	41 (43.2%)	54 (56.8%)	

As shown in Table 3, the thyrotropin levels of patients with NG-PTC were greater than those of patients with NG-nonPTC and only-NG. The thyrotropin level of patients with NG-PTC was 3.0 (1.3-62.0) µIU/mL, which was significantly greater than that of patients with only-NG (1.52 (0.3-5) µIU/mL) and that of patients with NG-nonPTC (1.46 (0.1-11.0) µIU/mL) (p = 0.02). The proportion of NG-PTC cases varied according to different thyrotropin levels. The percentage of NG-PTC patients with high-level TSH (74.2%) was greater than that of patients with low-level TSH (3.2%). There was a continuous increase in the rate of NG-PTC with increasing levels of thyrotropin (p < 0.001). The serum CEA levels of patients with NG-PTC (0.9 (0-2.35) ng/mL) were significantly greater than those of patients with NG-nonPTC (0 (0-0.58) ng/mL) and patients with non-NG (0 (0-0) ng/mL) (p = 0.018). However, the rates of NG-PTC, NG-nonPTC, and only-NG did not increase with increasing serum CEA levels. Specifically, in patients with NG-PTC, 40.0% had a serum CEA concentration ranging from 0 to 0 ng/mL, 20.0% had a serum CEA concentration ranging from 0.66 to 0.98 ng/mL, and 40.0% had a serum CEA concentration ranging from 1.37 to 9.48 ng/mL. The weight of patients with only-NG was lower than that of patients with NG-nonPTC or NG-PTC (p = 0.003). Further analysis revealed that the body mass index (BMI) of patients with only-NG was significantly lower than that of patients with NG-nonPTC or NG-PTC (p = 0.004).

**Table 3:** Difference of biochemical indexes for only nodular goitre (only-NG), nodular goitre coexistent with thyroid benign tumour (NG-nonPTC), and nodular goitre coexistent with papillary thyroid carcinomas (NG-PTC). *p < 0.05, **p < 0.001 TSH: median (min-max), CEA: median (p25-p75), were compared using the Kruskal-Wallis H test NG, nodular goitre: non-NG, without nodular goitre; TG, triglycerides; TC, total cholesterol; LDL, low-density lipoprotein; TSH, thyroid-stimulating hormone; Tg, thyroglobulin; CEA, carcinoembryonic antigen, PTC, papillary thyroid carcinoma; TPOAb: thyroid peroxidase antibodies; TGAb: thyroglobulin antibodies; BMI, body mass index

Variables	Only-NG, 51 (37.0%)	NG-nonPTC, 56 (40.5%)	NG-PTC, 31 (22.5%)	p-value
Female	40 (78.4%)	45 (80.4%)	24 (72.7%)	0.67
Age (years)	51.5 ± 12.6	49.5 ± 12.9	48.7 ± 12.5	0.58
Weight (kg)*	61.6 ± 10.4*	68.1 ± 8.7*	65.3 ± 10.1*	0.003
BMI (kg/m²)	23.8 ± 3.4*	26.1 ± 3.3*	24.8 ± 3.4*	0.004
<18.5	3.9%	0%	0%	<0.001
18.5-23.9	51.0%	30.4%	51.5%	
24-27.9	33.3%	41.1%	33.3%	
≥28	11.8%	28.6%	15.2%	
TG (mmol/L)	1.27 ± 0.10	1.59 ± 0.21	1.28 ± 0.11	0.11
TC (mmol/L)	4.87 ± 1.0	4.67 ± 0.80	4.62 ± 0.83	0.40
LDL (mmol/L)	3.20 ± 0.70	3.02 ± 0.71	3.06 ± 0.55	0.58
TSH (µIU/mL)*	1.52 (0.3-5.3)*	1.46 (0.1-11.0)*	3.0 (1.3-62.0)*	0.02
0.1-1.4	33.3%	48.2%	3.2%	<0.001
1.4-2.3	51.0%	25.0%	22.6%	
2.3-11.0	15.7%	26.8%	74.2%	
FT3 (pmol/L)	5.60 ± 1.02	5.45 ± 0.75	5.75 ± 1.0	0.58
FT4 (pmol/L)	18.9 ± 2.7	16.9 ± 1.4	17.9 ± 1.6	0.21
Tg (ng/mL)	34.56 ± 10.3	68.7 ± 13.2	47.6 ± 9.8	0.36
TGAb (IU/mL)	29.9 ± 8.0	26.5 ± 5.1	36.9 ± 12.8	0.86
TPOAb (IU/mL)	17.2 ± 1.2	27.2 ± 5.4	24.0 ± 4.5	0.47
CEA (ng/mL)*	0 (0-0)*	0 (0-0.58)*	0.90 (0-2.35)*	0.018

## Discussion

Importantly, thyroid carcinoma is the most common endocrine and neck tumour [6]. The incidence of thyroid carcinoma has increased in recent years. Many studies have shown a close relationship between NG and thyroid carcinoma. A large sample study in China showed that the incidence of differentiated thyroid carcinoma was significantly greater in patients with solitary NG than in those with MNG [7]. Consistent with the results of a previous study, our study showed that the incidence of thyroid carcinoma differed in patients with NG. In this study, the median age of patients who underwent NG was 48.2 years, which was also reported in other studies [7]. A large multicentre study in China showed that the median age of patients with NG was nearly 50 years, which was similar for male and female patients.

In our study, the rate of haemorrhage in the NG group was 29%. Some studies have shown that approximately 50% of thyroid nodules can cause spontaneous haemorrhage [8]. Spontaneous haemorrhage may lead to pain in the neck [9] and compress the trachea and oesophagus. Eventually, it causes difficulty breathing and even death. In the end, emergency surgery is important for spontaneous haemorrhage [10]. However, the mechanism of haemorrhage is unclear. The possible underlying biological mechanism may be as follows: the spongiform contents of thyroid nodules can provide rich nutrients for their growth. The non-smooth margin of the thyroid nodule and the oozing fluid that bleeds from the rupture of a weak vessel may be important reasons [11]. Therefore, positive and effective emergency surgery is very important for preventing haemorrhage from NG.

In our study, approximately 43.8% of the patients with thyroid carcinoma were diagnosed pathologically. Although NG is more likely to be associated with thyroid adenoma and thyroid cysts, up to 22.5% of thyroid carcinomas occur in patients with NG. In this study, there were more female NG patients with thyroid carcinoma than male NG patients, with a male-to-female ratio of 1:2.5. A large number of studies have shown that more than half of thyroid carcinomas occur in females [7,12]. One study showed that the rs944289 polymorphism, which is one of three single-nucleotide polymorphisms, had a significant relationship with the risk of thyroid carcinoma in females [13]. A greater understanding of the molecular mechanisms underlying sex differences in thyroid carcinoma pathogenesis could provide insights into diagnosis and treatment. A study showed that sex hormones play an important role in thyroid carcinoma pathogenesis [14]. Thyroid carcinoma is the fifth most common cancer in females but is not among the 15 most common cancers in males [15,16]. This difference in incidence rate may be because females have larger thyroid glands and more thyroid follicles than males. On the other hand, female sex hormones likely directly participate in the pathogenesis of NG. However, most studies have shown that benign thyroid tumours account for more than 90% of all thyroid nodules [12], and the cancer rate of NG is approximately 5%. Studies have shown that the pathogenesis of NG is similar to that of thyroid carcinoma. Research has shown that the disappearance of physiological stimulation of the thyroid gland, or the use of iodine, leads to thyroid fibrosis. The large fibres of the thyroid lead to NG over time. In the end, NG leads to deleterious mutations, cell death, and thyroid carcinoma [3]. Therefore, there is a greater incidence of thyroid carcinoma in the presence of NGs.

Genetics and the environment are essential for the pathogenesis of thyroid carcinoma, and the former is the most important factor [7]. Excessive imaging in medicine is one of the reasons for the increasing incidence of thyroid carcinoma. The most striking examples of radiation exposure are the accident at the Chernobyl nuclear reactor in 1986 and the Fukushima nuclear power plant in 2011. In the same thyroid follicle, while a thyrocyte may show an excessive mitotic response to proliferative stimuli, a neighbouring thyrocyte in the same follicle may have very low mitotic characteristics. One study showed that thyrotropin is the main mitotic factor [4]. A large number of studies have shown that a higher level of thyrotropin has a negative influence on thyroid carcinoma. Therefore, thyrotropin is closely related to NG and thyroid carcinoma. Kim et al. [17] investigated 1,638 patients and reported that the thyrotropin concentration was 2.1 ± 2.0 mU/L in thyroid carcinoma patients, which was greater than that in benign thyroid tumours. Further research revealed that the upper third of the normal range of thyrotropin and thyrotropin levels above the normal range were risk factors for thyroid carcinoma [17]. Rianto et al. [18] reported that the rate of thyroid malignancy in patients with high thyrotropin levels was approximately 65%, which was greater than that in patients with benign thyroid nodules and high thyrotropin levels (approximately 15%). Patients with high thyrotropin levels have an 8.42 times greater risk of developing malignant tumours than patients with low thyrotropin levels. These studies revealed the relationship between thyrotropin and thyroid carcinoma and provided evidence that high thyrotropin may promote thyroid carcinoma. Thyrotropin enhances adenylate cyclase activity, which also increases cyclic adenosine monophosphate production and cell growth through TSH receptors in vitro. The polymorphism of thymine nucleotides significantly increased the risk of thyroid carcinoma and NG [13]. In contrast, one study showed that rs965513, a thymine nucleotide polymorphism, is not significantly associated with the risk of thyroid carcinoma in East Asians [19]. Therefore, there are no consistent results on the relationship between rs965513 and thyroid carcinoma. In our study, the level of thyrotropin in the NG-PTC group was significantly greater than that in the simple NG group, and the proportion of the former increased significantly with increasing thyrotropin. Therefore, when we find that patients with NG have high thyrotropin levels, we need to be alert to the occurrence of thyroid carcinoma and increase the frequency of patient examination.

In our study, there was a significant difference in the CEA level between the NG group and the non-NG group. The risk of thyroid carcinoma from NG increases with the level of CEA. A higher level of CEA in patients with medullary thyroid carcinoma may be the reason for the transition from a normal thyroid to a malignant thyroid tumour [20]. CEA is a glycoprotein with a molecular weight of 200 kDa and is a component of the glycocalyx on the endoluminal side of the cell membrane of normal epithelial interstitial cells. One study showed that CEA participates in cell adhesion and may inhibit apoptosis, which causes normal thyroid tissue to become cancerous [21].

Limitations

The evidence of our study primarily relies on cross-sectional studies, which mainly describe a phenomenon without establishing causal relationships. Considering the limited number of subjects in this study, our results, however, need to be validated in a larger number of subjects, probably in a multicentre study.

## Conclusions

In our study, 22.5% of patients with NG also had PTC. This suggests that NGs more easily progress to thyroid carcinoma. Patients with NG and thyroid carcinoma have higher levels of thyrotropin and CEA compared to patients with NG alone. Therefore, higher levels of thyrotropin and CEA in NG are danger signals that increase the risk of thyroid carcinoma.
